# Predictive value of Altmetric score on citation rates and bibliometric impact

**DOI:** 10.1093/bjsopen/zraa039

**Published:** 2021-01-09

**Authors:** D B T Robinson, A G M T Powell, J Waterman, L Hopkins, O P James, R J Egan, W G Lewis

**Affiliations:** Health Education and Improvement Wales’ School of Surgery, Ty Dysgu, Nantgarw, UK; Division of Cancer and Genetics, Cardiff University, Heath Park, Cardiff, UK; Health Education and Improvement Wales’ School of Surgery, Ty Dysgu, Nantgarw, UK; Health Education and Improvement Wales’ School of Surgery, Ty Dysgu, Nantgarw, UK; Health Education and Improvement Wales’ School of Surgery, Ty Dysgu, Nantgarw, UK; Morriston Hospital, Cwmrhydceirw, Swansea, UK; Swansea University, Singleton Park, Sketty, Swansea, UK; Health Education and Improvement Wales’ School of Surgery, Ty Dysgu, Nantgarw, UK

## Abstract

**Background:**

Bibliometric and Altmetric analyses provide different perspectives regarding research impact. This study aimed to determine whether Altmetric score was associated with citation rate independent of established bibliometrics.

**Methods:**

Citations related to a previous cohort of 100 most cited articles in surgery were collected and a 3-year interval citation gain calculated. Citation count, citation rate index, Altmetric score, 5-year impact factor, and Oxford Centre for Evidence-Based Medicine levels were used to estimate citation rate prospect.

**Results:**

The median interval citation gain was 161 (i.q.r. 83–281); 74 and 62 articles had an increase in citation rate index (median increase 2.8 (i.q.r. –0.1 to 7.7)) and Altmetric score (median increase 3 (0–4)) respectively. Receiver operating characteristic (ROC) curve analysis revealed that citation rate index (area under the curve (AUC) 0.86, 95 per cent c.i. 0.79 to 0.93; *P* < 0.001) and Altmetric score (AUC 0.65, 0.55 to 0.76; *P* = 0.008) were associated with higher interval citation gain. An Altmetric score critical threshold of 2 or more was associated with a better interval citation gain when dichotomized at the interval citation gain median (odds ratio (OR) 4.94, 95 per cent c.i. 1.99 to 12.26; *P* = 0.001) or upper quartile (OR 4.13, 1.60 to 10.66; *P* = 0.003). Multivariable analysis revealed only citation rate index to be independently associated with interval citation gain when dichotomized at the median (OR 18.22, 6.70 to 49.55; *P* < 0.001) or upper quartile (OR 19.30, 4.23 to 88.15; *P* < 0.001).

**Conclusion:**

Citation rate index and Altmetric score appear to be important predictors of interval citation gain, and better at predicting future citations than the historical and established impact factor and Oxford Centre for Evidence-Based Medicine quality descriptors.

## Teaser

This study aimed to determine whether Altmetric score (AS) was associated with citation rate independent of established bibliometrics. Citations related to a previous cohort of 100 most cited articles (keyword surgery, 2018) were collected and a 3-year interval citation gain (iCG) calculated. Citation rate index (CRI) and AS appear to be important predictors of iCG, and better at predicting future citations than the historical and established impact factor (IF) and Oxford Centre for Evidence-Based Medicine quality descriptors.

## Resumen

**Antecedentes:**

Los análisis bibliométricos clásicos y alternativos (Altmetrics) brindan perspectivas diferentes sobre el impacto de la investigación. Este estudio tuvo como objetivo determinar si la puntuación Altmetric se asoció con la tasa de citación independiente de la bibliométrica estándar.

**Métodos:**

Se recopilaron las citaciones de una cohorte compuesta por los 100 artículos más citados en cirugía y se calculó el incremento de citaciones a intervalos de tres años. Se calcularon el número de citaciones, el índice de la tasa de citas, la puntuación Altmetric, el factor de impacto a los cinco años y los niveles de evidencia del Centro de Medicina basado en la Evidencia de la Universidad de Oxford.

**Resultados:**

La mediana del incremento del número de citaciones fue de 161 (rango intercuartílico, *interquartile range*, IQR 83-281), con 73 y 62 artículos que tuvieron un aumento del índice de la tasa de citas (mediana de incremento 2,8 (IQR -0,1-7,7)) y de la puntuación Altmetric (mediana de incremento 3 (IQR 0- 4)), respectivamente. El análisis ROC demostró que tanto el índice de tasa de citas (área bajo curva, *area under the curve*, AUC 0,86 (i.c. del 95%: 0,79-0,93), *P* < 0,001)) como la puntuación Altmetric (AUC 0,65 (i.c. del 95%: 0,55-0,76), *P* = 0,008)), se asociaron con el incremento de citas durante el periodo de estudio. Una puntuación Altmetric ≥ 2,0 se asoció con un mayor incremento de citaciones en el periodo analizado al dicotomizar la mediana del incremento de citas (razón de oportunidades, *odds ratio*, OR = 4,94; i.c. del 95%: 1,99-12,26, *P* = 0,001) y el cuartil superior (*upper quartile*, UQ; OR = 4,13, (1,60-10,66), *P* = 0,003). El análisis multivariable demostró que solo el índice de tasa de citaciones se asociaba de forma independiente con el incremento de citaciones durante el período cuando se dicotomiza en la mediana (OR 18,22, (6,70-49,55), *P* < 0,001) o en el UQ (OR 19,30, (4,23-88,15), *P* < 0,001)).

**Conclusión:**

Los índices de citación y la puntuación Altmetric parecen ser factores predictores de peso en el incremento del número de citaciones y son mejores para predecir las citaciones futuras que los factores de impacto histórico y establecido y los descriptores de calidad del CEBM de la Universidad de Oxford.

## Introduction

Academic reach and impact are sought after by author and journal editorial boards. Currently, the number of citations achieved by any given article is the most commonly used measure of academic reach, and the foundation of author Hirsch index (h-index) and journal impact factor (IF). In many countries, higher education institutions allocate funding according to academic outputs associated with journals held in high esteem on the basis of these traditional measures^1^^,^^2^.

Citations, however, take time to accumulate and other faster means of assessment have risen: alternative metrics, or Altmetrics. Altmetrics extend the concept of citation beyond mention in other scholarly articles by recording how often an article is downloaded, and applies to people, journals, books, data sets, presentations, videos, source code repositories, and web pages. The Altmetric score (AS) calculates impact based on diverse online research outputs, including social media, blogs, online news media, and online reference managers^3^. Consequently, the concept of impact remains as important and relevant a philosophical question as ever^3^^,^^4^.

Articles with a higher AS have been reported to be associated with higher citation counts^3^^,^^5^, but whether this symbolizes preliminary increased curiosity or sustained citation growth is unknown. The aim of this study was to identify the factors associated with citation accumulation, using a historical control cohort of the 100 most cited articles in surgery (2018)^3^. The hypothesis was that AS is directly related to prospective citations and an indicator of citation trajectory.

## Methods

Citation counts related to a previously reported cohort of 100 all-time most cited articles from a search performed in 2017 associated with the keyword ‘surgery’ were reanalysed^3^. Thomas Reuters’ Web of Science citation index was accessed again, providing a 3-year interval citation gain (iCG). Previously collected bibliometric data, including AS, citation count, citation rate index (CRI; total number of citations divided by number of years since article was published)^6^, 5-year IF, and Oxford Centre for Evidence-Based Medicine (OCEBM)^7^ quality of evidence levels were used to classify citation rate prediction.

### Altmetric scores

An AS is a weighted count of all of the mentions that Altmetric has tracked for an individual research output, and is designed to be an indicator of the degree of notice or attention an item has received. Scores are accumulated in an adjusted fashion to reflect the relative influence of different sources. For example, getting newspaper attention is more difficult than Twitter attention from a single individual; consequently, newspaper articles are more likely to receive attention than tweets and are thus allocated a higher contribution to a research output’s score. AS values are always whole numbers and, importantly, only the first mention from a source contributes to its accumulation. Some sources of attention will contribute less than 1 whole point to an output’s AS; in such instances, the AS is rounded up to the next whole number. The weighted scoring system can be found on the Altmetric^®^ website^8^.

### Web of Science category and quartile rank status

A journal’s IF category rank was recorded from Web of Science. A percentile rank (*z*) was calculated by dividing the rank (*x*) by the number of journals in a subject field (*y*), where *z* = *x*/*y*^9^. The percentile rank is categorized into four quartiles (Q1–4), with the most prestigious journals in the top quartile. Q1 journals are those belonging to the top 25 per cent, and Q4 are those within the bottom 25 per cent in a given subject field.

### Statistical analysis

All data were expressed as median (i.q.r.) and non-parametric statistical methods used throughout. The OCEBM scoring system for quality of evidence was reported in a Likert-scale format (level 1, highest level of evidence—systematic review or meta-analysis of RCTs; level 5, lowest level of evidence—expert opinion without critical appraisal)^7^. The Spearman correlation coefficient test was used to test the relationship between quality of evidence and iCG. The iCG was clustered into deciles to meet the test assumptions. Receiver operating characteristic (ROC) curve analysis was used to assessed the predictive value of continuous variables, with the primary outcome measure iCG dichotomized at the median and top quartile^10^. Univariable and multivariable logistic regression models were developed to identify independent associations with the primary outcome measures. Variables with *P* < 0.100 in univariable analysis were included in the multivariable analysis, using a backward conditional model. All statistical analysis was performed using SPSS^®^ version 25.0 (IBM, Armonk, New York, USA).

## Results

The total cumulative citations and iCG for all articles to date were 100 127 and 28 990 respectively. Ninety-eight articles were published in Q1 journals, one in a Q2 and one in a Q3 journal; there were no articles published in Q4 journals. Every article received more citations, with a median iCG of 161 (i.q.r. 83–281). Seventy-four articles had an increase in CRI (median gain 2.8 (–0.1 to 7.7)), it remained constant for one, and decreased for 25. AS increased for 62 articles (median gain 3 (0–4)), did not change for 36, and decreased for two. The top 10 articles related to the highest iCG can be found in *Table 1*^11–20^, and the full article list is available in *Table S1*. Of the original 10 most cited articles in 2017, eight maintained their top ten status 3 years later in terms of total citations.

**Table 1:** Top ten articles based on interval citation gain

Rank based on iCG	iCG	Reference	Title	Country	Year of publication	Original citation rank	Current citation rank
1	6046	Dindo *et al.*^11^	Classification of surgical complications: a new proposal with evaluation in a cohort of 6336 patients and results of a survey	Switzerland	2004	1	1
2	1868	Clavien *et al.*^12^	The Clavien–Dindo classification of surgical complications: 5-year experience	Switzerland	2009	5	2
3	1619	Norgren *et al.*^13^	Inter-society consensus for the management of peripheral arterial disease (TASC II)	Sweden	2007	25	3
4	1150	Norgren *et al.*^14^	Inter-society consensus for the management of peripheral arterial disease (TASC II)	Sweden	2007	8	4
5	981	Slim *et al.*^15^	Methodological index for non-randomized studies (minors): development and validation of a new instrument	France	2003	80	11
6	558	Lacroix *et al.*^16^	A multivariate analysis of 416 patients with glioblastoma multiforme: prognosis, extent of resection, and survival	USA	2001	11	8
7	552	Fong *et al.*^17^	Clinical score for predicting recurrence after hepatic resection for metastatic colorectal cancer: analysis of 1001 consecutive cases	USA	1999	2	5
8	488	Wente *et al.*^18^	Delayed gastric emptying (DGE) after pancreatic surgery: a suggested definition by the International Study Group of Pancreatic Surgery (ISGPS)	Germany	2007	42	18
9	487	Nashef *et al.*^19^	European system for cardiac operative risk evaluation (EuroSCORE)	England	1999	3	6
10	456	Rutherford *et al.*^20^	Recommended standards for reports dealing with lower extremity ischemia: revised version	USA	1997	4	7

iCG, Interval citation gain.

**Table 2:** Relationship between bibliometric variables, interval citation gain, and Altmetric score in predicting gains in citations.

	Score	AUC	*P*	Threshold
**Original AS**	0 (0–5; 0–53)	0.65 (0.55, 0.76)	0.008	1.5
**Original citation count**	573.5 (488.8–707.5; 446–5746)	0.65 (0.54, 0.75)	0.012	520.5
**Original CRI**	34.7 (26.3–45.9; 8.0–442)	0.86 (0.79, 0.93)	< 0.001	34.4
**Original 5-year IF**	4.9 (3.3–8.7; 1.4–8.7)	0.52 (0.41, 0.64)	0.680	n.a.*
**OCEBM level**	n.a.	0.46 (0.35, 0.58)	0.545	n.a.*

Values are median (i.q.r.; range). *Data assumptions for the Youden index were violated, so dichotomization points associated with original impact factor (IF) and quality of evidence were not considered. AUC, area under the curve; AS, Altmetric score; CRI, citation rate index; n.a., not available; OCEBM, Oxford Centre for Evidence-Based Medicine.

**Table 3:** Univariable and multivariable binary logistic regression analysis of factors associated with higher interval citation gain

	*n*	Univariable analysis				Multivariable analysis		
		Odds ratio	*P*			Odds ratio		*P*
**Median**								
RCT (no/yes)	87/13	1.19 (0.37, 3.84)	0.766					
Multicentre study (no/yes)	65/35	2.70 (1.15, 6.34)	0.023			1.48 (0.48, 4.59)		0.501
Primary institute appears more than once in top 100 (no/yes)	73/27	0.85 (0.35, 2.07)	0.726					
First or last author appears more than once in top 100 (no/yes)	82/18	3.16 (1.03, 6.96)	0.044			2.26 (0.54, 9.50)		0.265
OCEBM level (1/2/3/4/5)	13/19/3/46/15	0.84 (0.62, 1.14)	0.269					
Original AS ≥ 2* (low/high)	65/35	4.94 (1.99, 12.26)	0.001			2.46 (0.82, 7.40)		0.109
Original citation count ≥ 521* (low/high)	37/63	3.17 (1.35, 7.44)	0.008			1.84 (0.64, 5.28)		0.259
Original CRI ≥ 34.4* (low/high)	49/51	16.44 (6.02, 44.94)	< 0.001			18.22 (6.70, 49.55)		< 0.001
**Upper quartile**								
RCT (no/yes)	87/13	2.09 (0.62, 0.71)	0.237					
Multicentre study (no/yes)	65/35	1.67 (0.66, 4.22)	0.278					
Primary institute appears more than once in top 100 (no/yes)	73/27	1.34 (0.50, 3.60)	(0.565					
First or last author appears more than once in top 100 (no/yes)	82/18	3.06 (1.05, 8.94)	0.041			2.02 (0.59, 6.92)		0.266
OCEBM level (1/2/3/4/5)	13/19/3/46/15	0.90 (0.63, 1.27)	0.535					
Original AS ≥ 2* (low/high)	65/35	4.13 (1.60, 10.66)	0.003			2.07 (0.72, 6.00)		0.179
Original citation count ≥ 521* (low/high)	37/63	2.98 (1.01, 8.78)	0.048			1.24 (0.34, 4.54)		0.741
Original CRI ≥ 34.4* (low/high)	49/51	15.95 (3.45, 73.67)	< 0.001			19.30 (4.23, 88.15)		< 0.001

Values in parentheses are 95 per cent confidence intervals.

*Identified by receiver operating characteristic (ROC) curve analysis. OCEBM, Oxford Centre for Evidence-Based Medicine level; AS, Altmetric score; CRI, Citation Rate Index.

### Bibliometric factors associated with interval citation gain

Median article iCG when first or last authors featured more than once was 240 (i.q.r. 128–701.5), compared with 153.5 (75.5–257) (*P* = 0.011) when authors featured only once in the 100 most cited articles. Multicentre studies were not associated with higher iCGs than single-centre studies (median 183 (98–336) *versus* 143 (80–257); *P* = 0.209). Median iCG in relation to the country of the leading institute, OCEBM levels, research subject, disease category, and specialty are shown in *Figs S1–S5*. The highest and lowest iCG related to these variables were: country—median iCG 1084 (251–5002) for Switzerland *versus* 57 (42–134) for Japan; OCEBM evidence—291 (98–456) for level 5 *versus* 110 (83–135) for level 3; subject—456 (291–719) for epidemiology *versus* 101 (101–101) for nutrition; disease category—222 (166–321) for musculoskeletal *versus* 54 (54–54) for respiratory; and surgical specialty—235 (74–977) for vascular surgery *versus* 48 (48–48) for plastic surgery.

**Fig. 1:**
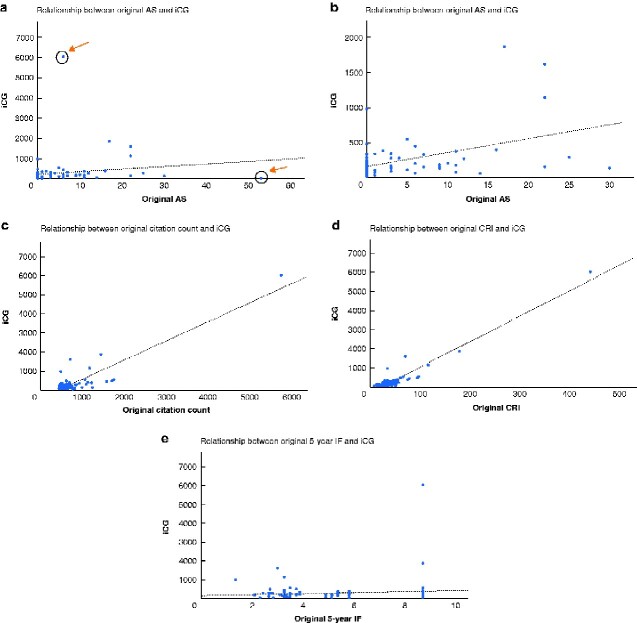
Scatter plots illustrating relationship between original variables and increase in number of citations over time Relationship between **a** original Altmetric score (AS) and interval citation gain (iCG) (*R*^2^ = 0.024, *P* = 0.121), **b** original AS and interval citation gain iCG with outliers (marked by arrows in **a**) removed (*R*^2^ = 0.199, *P* < 0.001), **c** original citation count and iCG (*R*^2^ = 0.797, *P* < 0.001), **d** original citation rate index (CRI) and iCG (*R*^2^ = 0.908, *P* < 0.001), and **e** original 5-year impact factor (IF) and iCG (*R*^2^ = 0.011, *P* = 0.311).

The relationship between original AS, number of citations, CRI, 5-year journal IF, and iCG is shown in *Fig. 1*. Linear regression analysis showed that original citation count (*R*^2^ = 0.797; 95 per cent c.i. 0.90 to 1.10; *P* < 0.001) and CRI (*R*^2^ = 0.908; 95 per cent c.i. 12.47 to 14.17; *P* < 0.001) were associated with iCG. Original AS (*R*^2^ = 0.024, 95 per cent c.i. –3.40 to 28.66; *P* = 0.121), 5-year journal IF (*R*^2^ = 0.011; 95 per cent c.i. –25.82 to 80.24; *P* = 0.311), and OCEBM level (*R*^2^ = 0.002; 95 per cent c.i. –124.50 to 78.45; *P* = 0.653) were not associated with iCG. When the two outliers were removed from the original AS analysis, AS (*R*^2^ = 0.199; 95 per cent c.i. 0.01 to 0.01; *P* < 0.001) was also associated with iCG (*Fig. 1b*).

### Creation of dichotomization thresholds for confounding adjustment

**Fig. 2:**
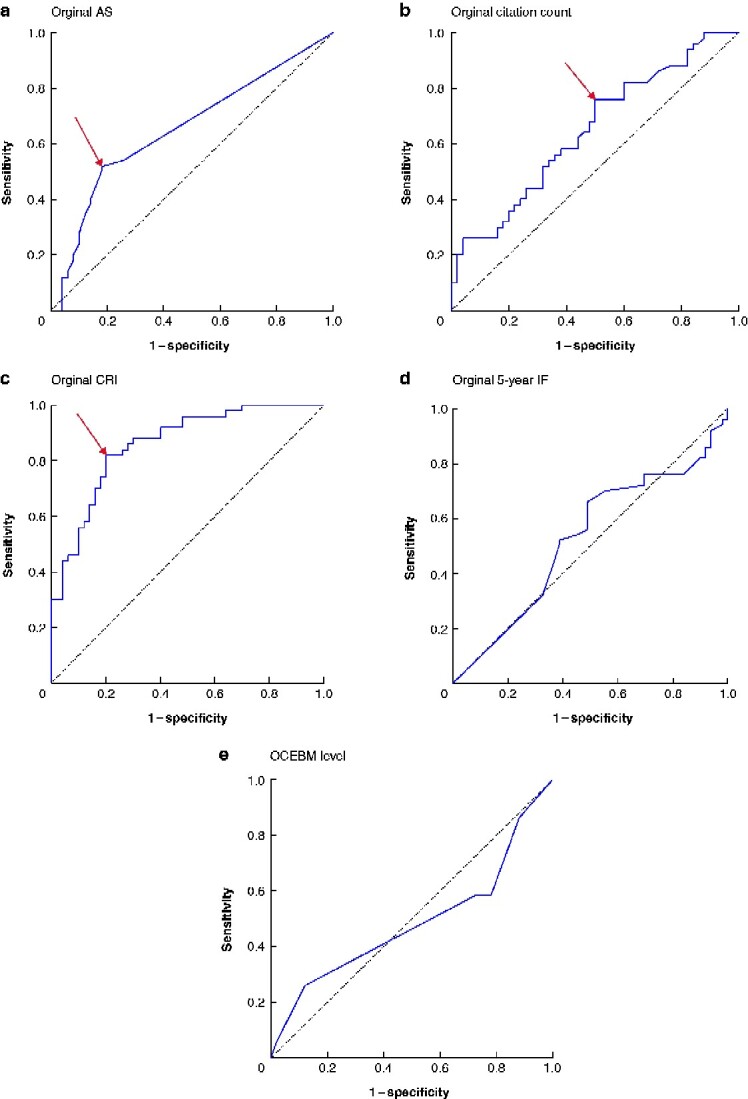
Receiver operating characteristic curves to identify critical thresholds for variables **a** Original Altmetric score (AS), **b** original citation count, **c** original citation rate index (CRI), **d** original 5-year impact factor (IF), and **e** Oxford Centre for Evidence-Based Medicine level. Arrows indicate the point taken to establish critical thresholds.

ROC curve analysis was used to identify possible critical thresholds for variables associated with higher iCG (*Fig. 2*). *Table 2* outlines the critical thresholds associated with higher iCG. No definitive critical thresholds were identified for 5-year IF (*Fig. 2d*) or OCEBM levels (*Fig. 2e*). The critical thresholds for original AS, citation count, and CRI were 1.5, 520.5, and 34.4 respectively. Articles with an AS of at least 2 (median iCG 209 (i.q.r. 143–393) *versus* 133 (71–234.5); *P* < 0.001), citation count 521 or more (192 (116–300) *versus* 120 (70–173); *P* = 0.004), and CRI at least 34.4 (250 (163–383) *versus* 95 (61–157); *P* < 0.001) were associated with higher median iCGs than those with values below these thresholds.

### Univariable and multivariable analysis of factors associated with above median and upper quartile of interval citation gain

Univariable and multivariable analyses of factors associated with iCG are shown in *Table 3*. In univariable analysis, multicentre studies (*P* = 0.023), articles with first or last author featured more than once in the 100 most cited articles (*P* = 0.044), an original AS of at least 2 (*P* = 0.001), original citation count 521 or more (*P* = 0.008), or original CRI at least 34.4 (*P* < 0.001) were associated with higher iCG dichotomized around the median. When dichotomized around the upper quartile value, articles where the first or last author featured more than once (*P* = 0.041), those with an original AS of at least 2 (*P* = 0.003), original citation count 521 or more (*P* = 0.048), or original CRI at least 34.4 (*P* < 0.001) were associated with higher iCG. In multivariable analysis, only CRI was associated with higher citation accrual when iCG was dichotomized around the median (odds ratio (OR) 18.22, 95 per cent c.i. 6.70 to 49.55; *P* < 0.001) or upper quartile (OR 19.30, 4.23 to 88.15; *P* < 0.001).

## Discussion

The principal finding of this study was that articles that amass citations at faster rates continue to grow their citations in an autonomous fashion that reflects the type of study, quality of evidence, affiliation of author or academic productivity. Articles with a CRI of at least 34.4 were 18 times more likely to accrue greater numbers of citations. AS was associated with a higher iCG; univariable analysis revealed that an AS of 2 or more conferred a fivefold multiplication factor with respect to citation accrual over a 3-year interval, but this was not verified by multivariable analysis.

Despite the widespread use of several impact metrics to gauge professional success, it remains unclear just how technological or scientific power evolves. Sinatra and colleagues^21^ quantified the changes in impact and productivity of an academic scientist throughout a career, and reported that impact, as measured by influential publications, was distributed randomly through any given sequence of publications. This random-impact rule allows the formulation of a stochastic model that disengages the effects of individual ability, productivity, and chance, implying the existence of universal patterns governing escalation of academic productivity. The model assigns a unique individual and stable parameter Q to each scientist, which accurately predicts the evolution of impact, from the h-index to cumulative citations, and independent acknowledgements such as prizes. This suggests that a scientist can influence an article’s impact, regardless of content, which is supported by the observation that authors with more than one entry in the 100 most cited list went on to accrue more interval citations, irrespective of academic affiliation^9–12^^,^^15^^,^^20–32^. Having multiple publications was not autonomous of CRI in predicting iCG on statistical modelling, supporting a close association between author influence and citation accrual. Certain authors, or teams of authors, appeared to be associated with a positive multiplication coefficient or constant which, when allied to a novel project, good fit, and timeliness, accelerated and boosted citation trajectory.

Wang and co-workers^33^ reported an arithmetically derived mechanical model which showed that ultimate article impact, characterized by total lifetime citations, was largely predicted by a single factor described as fitness. Fitness is the perceived novelty and relevance that an article attracts from the research community at large. This may explain why the Clavien–Dindo classification article^11^, which received the greatest number of citations and had the highest iCG in this study, was one of the highest ranking articles in bibliometric analyses of surgery^34^, visceral surgery^35^, and laparoscopic surgery^36^. When Wang *et al.*^33^ compared articles from journals with an impact factor of 3.26, 10.48, and 33.62, articles with similar fitness received initial citations in a journal-dependent manner, but ultimately received similar lifetime citation counts, which may help to clarify why journal IF did not predict citation count in the original 2018 study^3^, or iCG in the present study. The findings described by Wang and colleagues justify how proximity of citation gain, which augments journal IF, is supported by social media platforms. Whether the impact of social media simply shifts mean citation count or boosts total citation count and impact is unclear^33^.

With increasing competition for research funding, emphasis has migrated towards proving impact using bibliometric profiles and, historically, article citation count has always been synonymous with impact. Bibliometric use is supported by the present findings; in particular, CRI derived from citation count was associated independently with iCG. Academic reach is now facilitated by electronic devices, and journals have embraced this paradigm shift with a greater online presence. Zerrweck and colleagues^37^ reported that 31.5 per cent (51.8 per cent recreational use) of general surgeons, and 39.7 per cent (68.6 per cent recreational use) of bariatric surgeons used social media on a daily basis for academic purposes, making it possible for researchers to play an active role in dissemination of their academic outputs, likely amplifying citations. Alternative metrics are part of this era in measuring impact. It remains to be seen whether preprint article repositories, which enable early sharing of articles on social media, result in higher AS and citations, providing journal editors with additional and early impact metrics.

This study has a number of inherent limitations. Articles included were published at different times and were therefore placed at different stages of citation accrual. Original AS values were not recorded at identical follow-up times; nonetheless, AS and CRI were associated with a higher iCG, and were therefore irrespective of time lapsed. These bibliometric markers were strongly associated with citation accrual. All articles predate the founding of Altmetric scoring (2011) and the majority (89.0 per cent) predate the creation of Twitter. As such, conclusions regarding the impact of social media on AS cannot be established for this cohort, and future work should focus on prospectively collected serial measures of bibliometrics and AS over time using current articles. The 5-year IFs used in this study were acquired via the Web of Science database during the original data collection period in 2017. Some of these values may have fluctuated over time, having been higher or lower at the time of each paper’s release. The 100 most cited articles related to surgery were the focus, and the findings must therefore be interpreted with caution, as these findings may not be applicable to scientific articles published in other research arenas, or articles published in lower Q-score-rated journals given the preponderance of Q1 journals in this cohort. Citations carry inherent bias in the form of institutional or self-citation that cannot be identified from an online database search. The study also has a number of strengths. Article citations and AS values were recorded simultaneously and from a reputable database—Web of Science. The bibliometric analysis has statistical power, limiting confounding variables, and bias relating to time from publication.

Despite the perceived reciprocal association and attraction between high-quality research articles and journals with a higher IF, it appears that article fit, social media promotion, and author power are also predictors of prospective article impact and citation accrual. If any journal’s editorial strategy is to publish articles expected to acquire high citation numbers, early upload to one of the emerging repository websites may well develop as a preferred plan before official periodical submission. Such tactics would allow a period of attention, scrutiny, reflection, AS attainment, and early citation accrual. Such a strategy may be an improvement on the current peer review system before publication in a journal.

## Funding

Royal College of Surgeons England

Health Education and Improvement Wales

## Supplementary Material

zraa039_Supplementary_Data
