# Supplementary material for: Predictive value of Altmetric score on citation rates and bibliometric impact
Source: BJS Open. 2021 Jan 9;5(1):zraa039. doi: 10.1093/bjsopen/zraa039 (PMC7893457; doi:10.1093/bjsopen/zraa039)
Supplement: zraa039_Supplementary_Data [file zraa039_supplementary_data.docx]

**Table S1 100 ringfenced articles ranked based on interval citation gain (iCG)**

| **Rank based on iCG** | **iCG** | **Title** | **First author** | **Country** | **Year of publication** | **Original citation rank** | **Current citation rank** |
| --- | --- | --- | --- | --- | --- | --- | --- |
| 1 | 6046 | Classification of surgical complications - A new proposal with evaluation in a cohort of 6336 patients and results of a survey | D. Dindo | Switzerland | 2004 | 1 | 1 |
| 2 | 1868 | The Clavien-Dindo Classification of Surgical Complications Five-Year Experience | PA Clavien | Switzerland | 2009 | 5 | 2 |
| 3 | 1619 | Inter-society consensus for the management of peripheral arterial disease (TASC II) | L Norgren | Sweden | 2007 | 25 | 3 |
| 4 | 1150 | Inter-society consensus for the management of peripheral arterial disease (TASC II) | L Norgren | Sweden | 2007 | 8 | 4 |
| 5 | 981 | Methodological index for non-randomized studies (minors): development and validation of a new instrument | K Slim | France | 2003 | 80 | 11 |
| 6 | 558 | A multivariate analysis of 416 patients with glioblastoma multiforme: prognosis, extent of resection, and survival | M Lacroix | USA | 2001 | 11 | 8 |
| 7 | 552 | Clinical score for predicting recurrence after hepatic resection for metastatic colorectal cancer - Analysis of 1001 consecutive cases | Y. Fong | USA | 1999 | 2 | 5 |
| 8 | 488 | Delayed gastric emptying (DGE) after pancreatic surgery: a suggested definition by the International Study Group of Pancreatic Surgery (ISGPS). | MN Wente | Germany | 2007 | 42 | 18 |
| 9 | 487 | European system for cardiac operative risk evaluation (EuroSCORE) | SAM. Nashef | England | 1999 | 3 | 6 |
| 10 | 456 | Recommended standards for reports dealing with lower extremity ischemia: Revised version | RB. Rutherford | USA | 1997 | 4 | 7 |
| 11 | 404 | Virtual reality training improves operating room performance - Results of a randomized, double-blinded study | NE Seymour | USA | 2002 | 9 | 10 |
| 12 | 393 | The outcome and repair integrity of completely arthroscopically repaired large and massive rotator cuff tears. | LM Galatz | USA | 2004 | 27 | 19 |
| 13 | 383 | THE MESORECTUM IN RECTAL-CANCER SURGERY - THE CLUE TO PELVIC RECURRENCE | RJ Heald | England | 1982 | 7 | 9 |
| 14 | 349 | Evidence-based surgical care and the evolution of fast-track surgery | H Kehlet | Denmark | 2008 | 70 | 33 |
| 15 | 347 | Traumatic glenohumeral bone defects and their relationship to failure of arthroscopic Bankart repairs: significance of the inverted-pear glenoid and the humeral engaging Hill-Sachs lesion. | SS Burkhart | USA | 2000 | 69 | 34 |
| 16 | 345 | Objective structured assessment of technical skill (OSATS) for surgical residents | JA Martin | Canada | 1997 | 13 | 14 |
| 17 | 337 | Revision of the CEAP classification for chronic venous disorders: consensus statement. | B Eklof | Sweden | 2004 | 30 | 20 |
| 18 | 336 | Health-related quality of life in total hip and total knee arthroplasty - A qualitative and systematic review of the literature | O Ethgen | Belgium | 2004 | 41 | 22 |
| 19 | 331 | Operative versus nonoperative treatment for stage 0 distal rectal cancer following chemoradiation therapy: long-term results | A Habr-Gama | Brazil | 2004 | 86 | 45 |
| 20 | 300 | Cardiac-valve surgery - The French Correction | A Carpentier | France | 1983 | 10 | 12 |
| 21 | 299 | Effects of intravenous fluid restriction on postoperative complications: comparison of two perioperative fluid regimens: a randomized assessor-blinded multicenter trial | B Brandstrup | Denmark | 2003 | 40 | 25 |
| 22 | 299 | Defining risk levels in locally advanced head and neck cancers: a comparative analysis of concurrent postoperative radiation plus chemotherapy trials of the EORTC (#22931) and RTOG (# 9501). | J Bernier | Switzerland | 2005 | 78 | 48 |
| 23 | 291 | Multimodal strategies to improve surgical outcome | H Kehlet | Denmark | 2002 | 46 | 27 |
| 24 | 287 | Risk factors and outcome in European cardiac surgery: analysis of the EuroSCORE multinational database of 19030 patients. | F Rogues | France | 1999 | 14 | 15 |
| 25 | 281 | A prospective randomized trial comparing percutaneous local ablative therapy and partial hepatectomy for small hepatocellular carcinoma | MS Chen | China | 2006 | 48 | 30 |
| 26 | 279 | The Department of Veterans Affairs' NSQIP: the first national, validated, outcome-based, risk-adjusted, and peer-controlled program for the measurement and enhancement of the quality of surgical care. National VA Surgica | SF Khuri | USA | 1998 | 15 | 17 |
| 27 | 277 | Complications of iliac crest bone graft harvesting. | ED Arrington | USA | 1996 | 32 | 24 |
| 28 | 257 | Pathological features of rectal cancer after preoperative radiochemotherapy | O Dworak | Germany | 1997 | 58 | 46 |
| 29 | 257 | Arthroscopic repair of full-thickness tears of the supraspinatus: does the tendon really heal? | P Boileau | France | 2005 | 94 | 59 |
| 30 | 250 | Consensus-based method for risk adjustment for surgery for congenital heart disease. | KJ Jenkins | USA | 2002 | 61 | 49 |
| 31 | 246 | Proposed classification of complications of surgery with examples of utility in cholecystectomy | PA Clavien | Canada | 1992 | 39 | 32 |
| 32 | 235 | The results of repair of massive tears of the rotator cuff | C Gerber | Switzerland | 2000 | 90 | 63 |
| 33 | 234 | Rescue surgery for unresectable colorectal liver metastases downstaged by chemotherapy: a model to predict long-term survival | R Adam | France | 2004 | 23 | 21 |
| 34 | 224 | An analysis of the problem of biliary injury during laparoscopic cholecystectomy | SM Strasberg | USA | 1995 | 35 | 28 |
| 35 | 209 | Outcomes of microfracture for traumatic chondral defects of the knee: average 11-year follow-up | JR Steadman | USA | 2003 | 62 | 55 |
| 36 | 209 | The TME trial after a median follow-up of 6 years: increased local control but no survival benefit in irradiated patients with resectable rectal carcinoma | KCMJ Peeters | The Netherlands | 2007 | 76 | 62 |
| 37 | 205 | Determining in vivo biomechanical properties of the cornea with an ocular response analyzer. | DA Luce | USA | 2005 | 57 | 52 |
| 38 | 203 | Rectal cancer: the Basingstoke experience of total mesorectal excision, 1978-1997 | RJ Heald | England | 1998 | 22 | 23 |
| 39 | 193 | Randomized clinical trial of virtual reality simulation for laparoscopic skills training | TP Grantcharov | Denmark | 2004 | 52 | 51 |
| 40 | 192 | Effect of surgical margin status on survival and site of recurrence after hepatic resection for colorectal metastases | TM Pawlick | USA | 2005 | 63 | 57 |
| 41 | 186 | Why are total knee arthroplasties failing today? | PF Sharkey | USA | 2002 | 53 | 53 |
| 42 | 183 | Adjuvant radiotherapy and 5-fluorouracil after curative resection of cancer of the pancreas and periampullary region: phase III trial of the EORTC gastrointestinal tract cancer cooperative group. | JH Klinkenbijl | The Netherlands | 1999 | 28 | 31 |
| 43 | 180 | Autologous chondrocyte implantation compared with microfracture in the knee - A randomized trial | G Knutsen | Norway | 2004 | 34 | 41 |
| 44 | 177 | Fecal Incontinence Quality of Life Scale: quality of life instrument for patients with fecal incontinence | TH Rockwood | USA | 2000 | 60 | 61 |
| 45 | 175 | Comparisons of outcome and prognostic features among histologic subtypes of renal cell carcinoma | JC Cheville | USA | 2003 | 71 | 65 |
| 46 | 171 | The importance of surgeon experience for clinical and economic outcomes from thyroidectomy | JA Sosa | USA | 1998 | 88 | 71 |
| 47 | 167 | Gut hormone profiles following bariatric surgery favor an anorectic state, facilitate weight loss, and improve metabolic parameters | CW le Roux | UK | 2006 | 92 | 76 |
| 48 | 163 | Effect of laparoscopic Roux-En Y gastric bypass on type 2 diabetes mellitus | PR Schauer | USA | 2003 | 44 | 50 |
| 49 | 162 | Clinical practice guideline: adult sinusitis | RM Rosenfeld | USA | 2007 | 87 | 72 |
| 50 | 161 | Metal-on-metal bearings and hypersensitivity in patients with artificial hip joints. A clinical and histomorphological study. | HG Willert | Germany | 2005 | 54 | 56 |
| 51 | 160 | Ileal pouch-anal anastomoses complications and function in 1005 patients. | VW Fazio | USA | 1995 | 29 | 38 |
| 52 | 160 | Continuous intravenous insulin infusion reduces the incidence of deep sternal wound infection in diabetic patients after cardiac surgical procedures | AP Furnary | USA | 1999 | 49 | 54 |
| 53 | 159 | The treatment of soft-tissue sarcomas of the extremities - prospective randomized evaluations of (1) limb-sparing surgery plus radiation-therapy compared with amputation and (2) the role of adjuvant chemotherapy | SA Rosenberg | USA | 1982 | 37 | 44 |
| 54 | 155 | The general rules for the gastric-cancer study in surgery and pathology .1. clinical classification | T KAJITANI | Japan | 1981 | 83 | 70 |
| 55 | 152 | Laparoscopic versus open subtotal gastrectomy for distal gastric cancer: five-year results of a randomized prospective trial | CGS Huscher | Italy | 2005 | 95 | 79 |
| 56 | 146 | An analysis of blood management in patients having a total hip or knee arthroplasty | Be Bierbaum | USA | 1999 | 91 | 78 |
| 57 | 145 | Autologous osteochondral mosaicplasty for the treatment of full-thickness defects of weight-bearing joints - Ten years of experimental and clinical experience | L Hangody | Hungary | 2003 | 98 | 82 |
| 58 | 143 | Surgery decreases long-term mortality, morbidity, and health care use in morbidly obese patients. | NV Christou | Canada | 2004 | 31 | 43 |
| 59 | 138 | The international-cooperative-study-on-the-timing-of-aneurysm-surgery .1. overall management results | NF Kassell | USA | 1990 | 6 | 13 |
| 60 | 137 | Surgical resection and radiolocalization of the sentinel lymph-node in breast-cancer using a gamma-probe | DN Krag | USA | 1993 | 12 | 16 |
| 61 | 136 | Testing technical skill via an innovative ''bench station'' examination | R Reznick | Canada | 1997 | 64 | 68 |
| 62 | 133 | experience with 1509 patients undergoing thoracoabdominal aortic operations | LG Svensson | USA | 1993 | 26 | 42 |
| 63 | 130 | Chronic electrical stimulation of the ventralis intermedius nucleus of the thalamus as a treatment of movement disorders. | AL Benabid | France | 1996 | 33 | 47 |
| 64 | 129 | Complications of transsphenoidal surgery: results of a national survey, review of the literature, and personal experience | I Ciric | USA | 1997 | 73 | 73 |
| 65 | 126 | Reporting standards in venous disease - an update | JM Porter | USA | 1995 | 21 | 29 |
| 66 | 120 | Rates of complications and death after pancreaticoduodenectomy: risk factors and the impact of hospital volume | DJ Gouma | The Netherlands | 2000 | 93 | 87 |
| 67 | 117 | Two internal thoracic artery grafts are better than one | BW Lytle | USA | 1999 | 81 | 80 |
| 68 | 116 | Relevant prognostic factors in gastric cancer: ten-year results of the German Gastric Cancer Study | JR Siewert | Germany | 1998 | 24 | 37 |
| 69 | 110 | Hazards of postoperative atrial arrhythmias | LL Creswell | USA | 1993 | 66 | 75 |
| 70 | 101 | Prognostic nutritional index in gastrointestinal surgery | GP Buzby | USA | 1980 | 43 | 60 |
| 71 | 98 | Outcomes after laparoscopic Roux-en-Y gastric bypass for morbid obesity | PR Schauer | USA | 2000 | 20 | 35 |
| 72 | 98 | ASGE/SAGES working group on natural orifice translumenal endoscopic surgery - October 2005 | D Rattner | USA | 2006 | 77 | 84 |
| 73 | 95 | Ten-year experience with 733 pancreatic resections: changing indications, older patients, and decreasing length of hospitalization | JH Balcom | USA | 2000 | 85 | 88 |
| 74 | 86 | Long-term survival after curative resection for pancreatic ductal adenocarcinoma. Clinicopathologic analysis of 5-year survivors | KC Conlon | USA | 1996 | 82 | 89 |
| 75 | 84 | Resection of nonresectable liver metastases from colorectal cancer after neoadjuvant chemotherapy | H Bismuth | France | 1996 | 38 | 58 |
| 76 | 82 | Electrothrombosis of saccular aneurysms via endovascular approach .2. preliminary clinical-experience | G Gugliemi | Italy | 1991 | 16 | 26 |
| 77 | 78 | Surgery without scars - Report of transluminal cholecystectomy in a human being | J Marescaux | France | 2007 | 68 | 83 |
| 78 | 76 | Laparoscopic versus open gastric bypass: a randomized study of outcomes, quality of life, and costs | NT Nguyen | USA | 2001 | 50 | 69 |
| 79 | 74 | Radiofrequency ablation of unresectable primary and metastatic hepatic malignancies: results in 123 patients. | SA Curley | USA | 1999 | 19 | 39 |
| 80 | 74 | Laparoscopic gastric bypass, Roux-en-Y- 500 patients: technique and results, with 3-60 month follow-up | AC Wittgrove | USA | 2000 | 74 | 90 |
| 81 | 72 | An analysis of 412 cases of hepatocellular carcinoma at a western center | YM Fong | USA | 1999 | 56 | 74 |
| 82 | 72 | Development and implementation of intraoperative magnetic resonance imaging and its neurosurgical applications | PM Black | USA | 1997 | 75 | 92 |
| 83 | 70 | Total mesorectal excision in the operative treatment of carcinoma of the rectum | WE Enker | USA | 1995 | 67 | 85 |
| 84 | 70 | Extension of the frontiers of surgical indications in the treatment of liver metastases from colorectal cancer - Long-term results | M Minagawa | Japan | 2000 | 99 | 96 |
| 85 | 68 | Pathophysiology of syringomyelia associated with chiari i malformation of the cerebellar tonsils - implications for diagnosis and treatment | EH Oldfield | USA | 1994 | 97 | 97 |
| 86 | 66 | Leksells posteroventral pallidotomy in the treatment of parkinsons-disease | LV Laitinen | Sweden | 1992 | 18 | 36 |
| 87 | 63 | Inflammatory response to cardiopulmonary bypass | J Butler | England | 1993 | 47 | 67 |
| 88 | 63 | Prevention of postoperative abdominal adhesions by a sodium hyaluronate-based bioresorbable membrane: a prospective, randomized, double-blind multicenter study. | JM Becker | USA | 1996 | 89 | 95 |
| 89 | 59 | The International Cooperative Study on the Timing of Aneurysm Surgery. Part 2: Surgical results. | NF Kassell | USA | 1990 | 45 | 66 |
| 90 | 56 | Cytokine patterns in patients after major vascular surgery, hemorrhagic shock, and severe blunt trauma. Relation with subsequent adult respiratory distress syndrome and multiple organ failure. | RMH Roumen | The Netherlands | 1993 | 84 | 93 |
| 91 | 54 | Bilateral pneumectomy (volume reduction) for chronic obstructive pulmonary disease | JD Cooper | USA | 1994 | 51 | 77 |
| 92 | 48 | Validation of the accuracy of intraoperative lymphatic mapping and sentinel lymphadenectomy for early-stage melanoma - A multicenter trial | DL Morton | USA | 1999 | 65 | 91 |
| 93 | 46 | Functional endoscopic sinus surgery - theory and diagnostic evaluation | DW Kennedy | USA | 1985 | 100 | 99 |
| 94 | 43 | Oesophageal squamous cell carcinoma. I. A critical review of surgery | R Earlam | Japan | 1980 | 17 | 40 |
| 95 | 41 | Progress in gastric-cancer surgery in japan and its limits of radicality | K Maruyama | Japan | 1987 | 55 | 81 |
| 96 | 41 | Hypothermia - its possible role in cardiac surgery - an investigation of factors governing survival in dogs at low body temperatures | WG Bigelow | Canada | 1950 | 59 | 86 |
| 97 | 33 | Sentinel lymph node biopsy in breast cancer: guidelines and pitfalls of lymphoscintigraphy and gamma probe detection | P Borgstein | The Netherlands | 1998 | 72 | 94 |
| 98 | 32 | Guglielmi detachable coil embolization of acute intracranial aneurysm: perioperative anatomical and clinical outcome in 403 patients. | F Vinuela | USA | 1997 | 36 | 64 |
| 99 | 27 | AneuRx stent graft versus open surgical repair of abdominal aortic aneurysms: Multicenter prospective clinical trial | CK Zarins | USA | 1999 | 96 | 100 |
| 100 | 26 | Guidelines for reporting morbidity and mortality after cardiac valvular operations | LH Edmunds | USA | 1996 | 79 | 98 |

**Fig. S1** **Median interval citation gain (iCG) related to the country of the leading institute of the study**

**Fig. S2** **Median interval citation gain (iCG) related to the Oxford Centre for Evidence Based Medicine level**

Four studies were omitted as they were training based and therefore did not fit into any of the evidence categories.

**Figure S3** **Median interval citation gain (iCG) related to the subject of the study**

**Figure S4** **Median interval interval citation gain (iCG) related to disease category**

Four studies were omitted as they were training based studies and therefore do not cover diseases.

**Fig. S5** **Median interval citation gain (iCG) related to specialty**

Seven studies were omitted as they were either applicable to multiple surgical specialities or were not speciality specific
